# Grading of carotid artery stenosis with computed tomography angiography: whether to use the narrowest diameter or the cross-sectional area

**DOI:** 10.1007/s13244-018-0622-5

**Published:** 2018-05-24

**Authors:** Kristina Samarzija, Petar Milosevic, Zoran Jurjevic, Emilija Erdeljac

**Affiliations:** 1 Department of Radiology, General Hospital Karlovac, Karlovac, Croatia; 2 Department of Vascular Surgery, General Hospital Karlovac, Karlovac, Croatia; 3 Department of Neurology, General Hospital Karlovac, Karlovac, Croatia

**Keywords:** Carotid artery stenosis, CT angiography, Color Doppler ultrasonography, Atherosclerosis, Medical imaging

## Abstract

**Objectives:**

To compare the estimation of carotid artery stenosis by computed tomography angiography (CTA) based on cross-sectional area versus the smallest diameter measurement, and test the accuracy of both CTA measurements using color Doppler ultrasonography (CDUS) as a reference method.

**Methods:**

For 113 carotid arteries with stenosis ≥50% we analysed the differences in the estimated stenosis level between both CTA methods and CDUS using the Bland-Altman approach. Further, we calculated sensitivity, specificity and plotted receiver operating characteristic (ROC) curves for both CTA methods.

**Results:**

The mean difference between CDUS and CTA (area) measurements was −0.4% (*p* = 0.68); between CDUS and CTA (diameter), 20.7% (*p* < 0.001); and between CTA (area) and CTA (diameter), 21.2% (*p* < 0.001). Sensitivity and specificity for the CTA (area) method were 81% and 77%, and for CTA (diameter) were 23% and 100%. The area under the curve (AUC) for CTA (diameter) was 0.62 (0.57, 0.66), and for CTA (area) 0.79 (0.71–0.87). The equality test for the two AUCs was <0.0001.

**Conclusions:**

CTA (diameter)-based measurements significantly underestimated the degree of carotid stenosis. We recommend the CTA (area) method because of its higher predictive power for a correct stenosis classification and a lack of significant difference in the estimated stenosis level, compared to CDUS.

**Main messages:**

• *Cross-sectional area measurement considers asymmetric shape of the residual vessel lumen.*

• *CTA (diameter) method on average significantly underestimates the true level of stenosis.*

• *CTA (area) method correctly classifies vessels needing surgical intervention.*

## Introduction

Stroke is one of the leading causes of death in developed countries. Atherosclerotic stenoses of extracranial carotid arteries are the major risk factor for the development of ischemic stroke. The largest clinical studies, the North American Symptomatic Carotid Endarterectomy Trial (NASCET) and the European Symptomatic Carotid Surgery Trial (ESCT), have shown a significant benefit of carotid endarterectomy in patients with severe carotid stenosis (70–99%) in reducing the risk of stroke [1, 2]. Therefore, accurate measurement of stenosis is of great importance in identifying patients requiring surgery. Today, various diagnostic modalities are available for evaluation of carotid artery disease: color Doppler ultrasonography (CDUS), computed tomography angiography (CTA), magnetic resonance angiography (MRA) and intra-arterial digital subtraction angiography (DSA) [3]. All these modalities have their advantages and disadvantages. The preferred method is most often specific to a single institution and usually depends on available equipment and personnel competences. DSA is still considered as a gold standard in the assessment of stenosis, but because of the stroke risk, patient’s discomfort and high cost, it is increasingly being replaced by noninvasive techniques. In our institution, CDUS is used as an initial diagnostic tool with CTA as additional imaging for preoperative evaluation. The use of different imaging modalities introduces disagreement in the assessment of the degree of carotid stenosis and leads to a difference of opinion as to which method is more accurate. In this study we compared the results in estimation of carotid stenosis obtained from CTA using two related features: the cross sectional area and the smallest diameter of the vessel lumen, and tested the accuracy of both CTA measurements using CDUS as a reference method.

## Materials and methods

### Study population

We made a retrospective analysis of patients who underwent both CDUS and CTA examinations of extracranial carotid arteries in our institution in the period from February 2014 to July 2015. All patients underwent CDUS and CTA examinations as part of the institutional routine diagnostic workup in evaluation of carotid stenosis and gave written informed consent for CTA. The study was performed with institutional medical ethics committee approval, which established that it was in compliance with ethical standards and that informed patient consent for inclusion in this retrospective study was not required.

The examinations were performed in patients with neurological symptomatology and in asymptomatic patients with risk factors for atherosclerosis or other known atherosclerotic disease (coronary or peripheral arterial disease). Patients with previous endarterectomy or endovascular treatment were excluded from the analysis.

A total of 91 patients were included in the study, 47 male and 44 female. The mean age was 71 years (range 47–89 years). The mean time interval between the CDUS and CTA examinations was 56 days (not longer than 6 months). The stenoses were classified into moderate (50–69%) and severe (70–99%) and were evaluated separately.

### Color Doppler ultrasonography

CDUS examinations were performed using a ProSound Alpha 7 ultrasound system (Aloka) with a 7.5 MHz linear transducer, by five radiologists with a minimum of 4 years of experience in ultrasound diagnostics. Grading of stenosis was performed according to the good-quality criteria recommended by the Society of Radiologists in Ultrasound Consensus Conference in 2003, with peak systolic velocity (PSV) of 125 cm/s as a threshold for 50% stenosis and 230 cm/s for 70% stenosis [4]. An end-diastolic velocity and ICA/CCA ratio were used as additional parameters.

### Computed tomography angiography

**Fig. 1:**
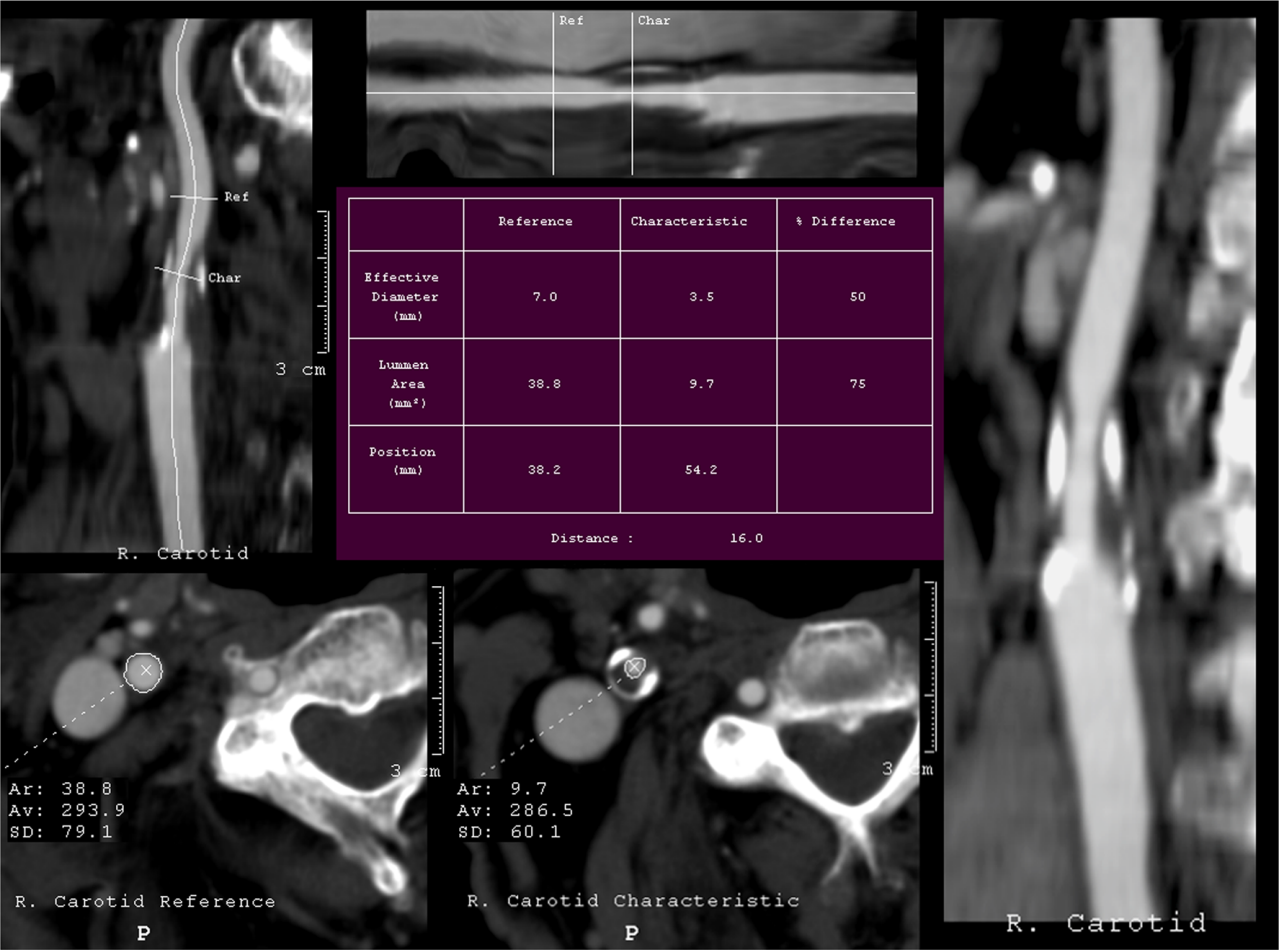
Automated quantification of an eccentric right carotid artery stenosis on CTA. Percentage of stenosis is calculated by electronically outlining the residual and the normal vessel lumen in a plane perpendicular to the longitudinal axis of the vessel. In this example, the area of the residual lumen is 9.7 mm^2^, the area of the normal lumen is 38.8 mm^2^, resulting in 75% stenosis. The residual lumen diameter is 3.5 mm, the normal lumen diameter is 7.0 mm, resulting in 50% stenosis

All CTA examinations were performed on a four-slice Mx8000 Quad CT scanner (Philips Medical Systems), with postprocessing on a Philips workstation MxView, by four radiologists with 3 to 9 years of experience in reading CTA studies. Examinations were done using standard protocols with a section thickness of 1.2 mm (1 mm collimation) and a reconstruction interval of 0.6 mm (50% overlapping). The CT scans covered the range between the aortic arch and the level of the circle of Willis. A total of 100 ml contrast agent (Xenetix 350) was injected in the antecubital vein at a flow rate of 4 ml/s. To optimize the acquisition delay, bolus triggering (Bolus pro ultra) was used with region of interest (ROI) placed in the aortic arch. Postprocessing images were created with multi-planar reformation (MPR), volume rendering (VR) and curved planar reformation (CPR) techniques. Quantification of stenosis was obtained according to NASCET criteria using a distal normal poststenotic segment as a referential site for comparison with the segment of maximal stenosis [5]. For stenosis quantification, an automated method was used, because it is, in our opinion, faster and easier, less operator dependent and more reproducible than manual methods. Calculation of stenosis was made using the Advanced Vessel Analysis (AVA) programme on a dedicated CT workstation with commercially available postprocessing software (Extended Brilliance Workspace version R.1.0.91, Philips Medical Systems), that evaluated the degree of stenosis in terms of the narrowest diameter and the cross sectional area at the point of maximum stenosis (Fig. 1).

### Statistical analysis

For each vessel we calculated differences in the estimated level of stenosis from all three possible pairs of methods (CDUS – CTA [area], CDUS – CTA [diameter] and CTA [area] – CTA [diameter]) and visually presented empirical distribution of these differences. We further tested, using T or Wilcoxon tests, whether any of these distributions had a central tendency at 0. Then we generated Bland-Altman (BA) plots for each pair of measurements, separately in the group of 50–69% stenosis and in the group of 70–99% stenosis. We further calculated sensitivity, specificity, positive predictive value (PPV) and negative predictive value (NPV) for both CTA methods. To visualize and test the predictive power of the two CTA methods with respect to binary classifications (70–99% stenosis that need surgical intervention and 50–69% stenosis without that need) we plotted receiver operating characteristic (ROC) curves and calculated the area under the curve (AUC) with a 95% confidence interval. We tested the difference with respect to the null hypothesis of no difference between these ROC curves up to the level of 0.05.

## Results

**Fig. 2:**
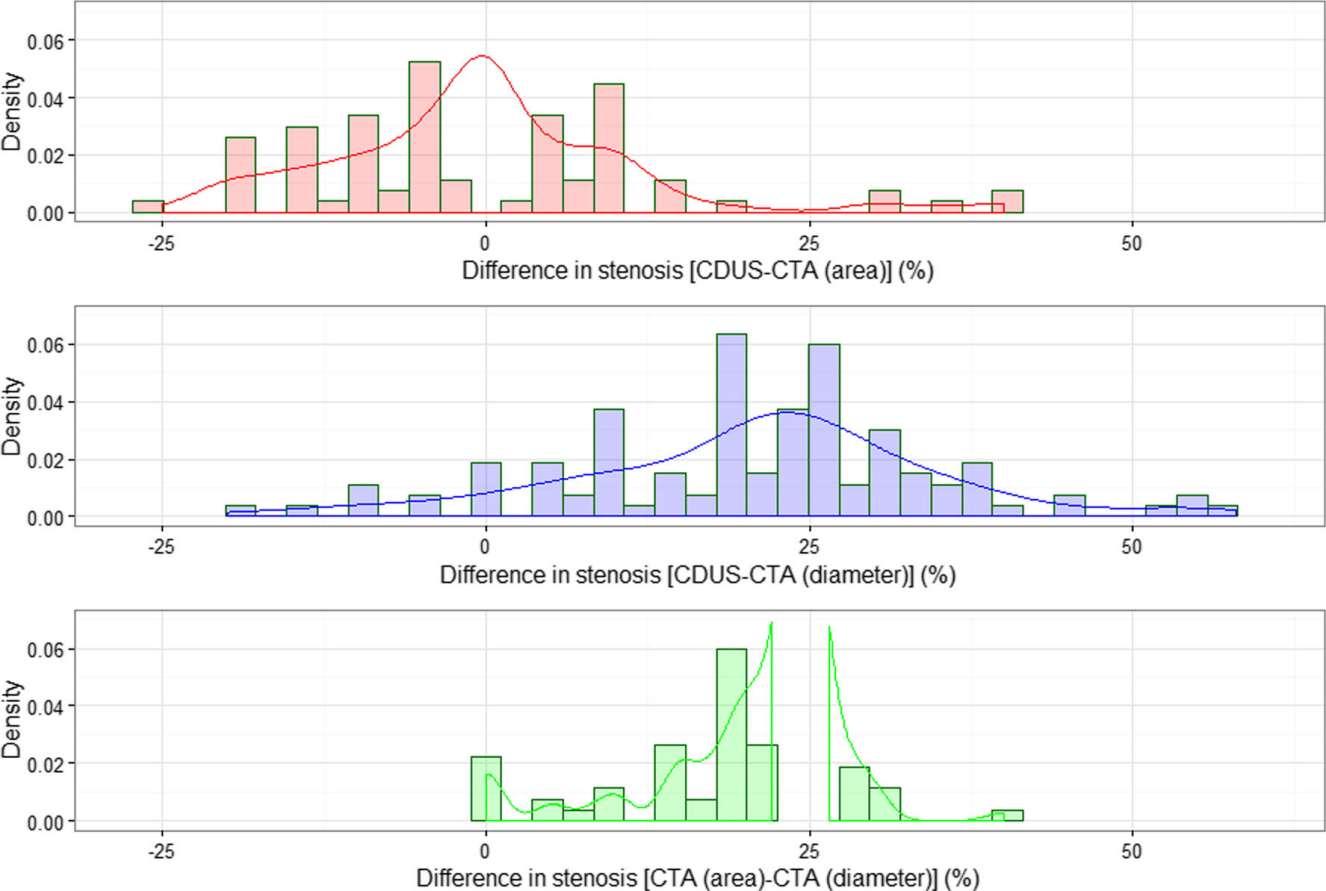
Empirical distributions of differences in stenosis measurements from three pairs of methods

Over a period of 17 consecutive months (February 2014 to July 2015), a total of 182 carotid arteries in 91 patients were imaged. Carotid stenosis of 50% or greater, diagnosed by ultrasound, were present in 113 of these and only those 113 stenotic carotid arteries were used for analysis in this study. There were 44 vessels in the group of moderate (50–69%) stenosis and 69 vessels in the group of severe (70% –99%) stenosis. The most commonly measured stenosis by CDUS was 70% (28% of all stenoses was estimated to 70%). Average stenosis measured by the CDUS method was 67.9% with a median of 70%. Among measurements by the CTA (area) method, average stenosis was 68.4% with a median value of 70%, also. Measurements from CTA (diameter) averaged at 47.2%, with a median of 45%. Figure 2 shows empirical distributions of differences in the estimated level of stenosis calculated from the three pairs of methods [CDUS – CTA (area), CDUS – CTA (diameter) and CTA (area) – CTA (diameter)].

Table 1 presents means and medians of differences in stenosis with the corresponding *p*-values from the test of the hypothesis that the central tendency of their distribution is 0.

**Table 1:** Location and spread parameters of distribution of differences in stenosis measurements [*p*-values from Student’s *t*-test and Wilcoxon rank sum test (in parenthesis)]

	All stenosis			Stenosis 50–69%			Stenosis 70–99%		
	Mean (SD)	Median (IQR)	*p*-value	Mean (SD)	Median (IQR)	*p*-value	Mean (SD)	Median (IQR)	*p*-value
CDUS − CTA (area)	−0.4 (11.8)	0 (−5, 5)	0.68 (0.33)	−4.5 (10.1)	−2.5 (−12.5, 0)	0.005 (0.004)	2.1 (12.0)	0 (−5, 10)	0.14 (0.22)
CDUS −CTA (diameter)	20.7 (14.2)	22 (10, 28)	< 0.001 (<0.001)	17.3 (9.9)	20 (10,24)	< 0.001 (<0.001)	22.9 (16.0)	25 (20, 32)	< 0.001 (<0.001)
CTA (area) − CTA(diameter)	21.2 (7.1)	24 (20, 25)	< 0.001 (<0.001)	21.7 (4.5)	23 (20,25)	< 0.001 (<0.001)	20.8 (8.4)	24 (18, 25)	< 0.001 (<0.001)

Figure 2 and the first row of Table 1 show location and tests of differences for all vessels in the sample. The central tendency of the distribution of differences between CDUS and CTA (area) measurements is around 0, with a mean of −0.4% and median of 0. In contrast, each of the two pairs involving CTA (diameter) measurements are centered around 20%, with both tests of location (mean and median) yielding significant *p*-values.

When the same analysis was performed separately within the moderate and severe stenosis groups, the results were similar. Both of the pairs that involved CTA (diameter) stenosis measurements tested significantly when the central tendency of the distribution of differences was assumed to be 0 under the null hypothesis. The bias was between 17.3% and 22.9% as measured by the mean (see Table 1), while it was even larger when the median was taken to mark the central tendency of the distribution. For vessels in the 50–69% stenosis group, the CTA (area) method slightly, but significantly, overestimated true stenosis, with a mean difference of −4.5%. Although both mean and median were significantly different from 0 for this group of stenosis, *p*-values were not as small as those from testing differences between CDUS and CTA (diameter). For the severe stenosis group (upwards of 70%), the mean difference between CDUS and CTA (area) was around 2% and failed to test significantly different from 0.

**Fig. 3:**
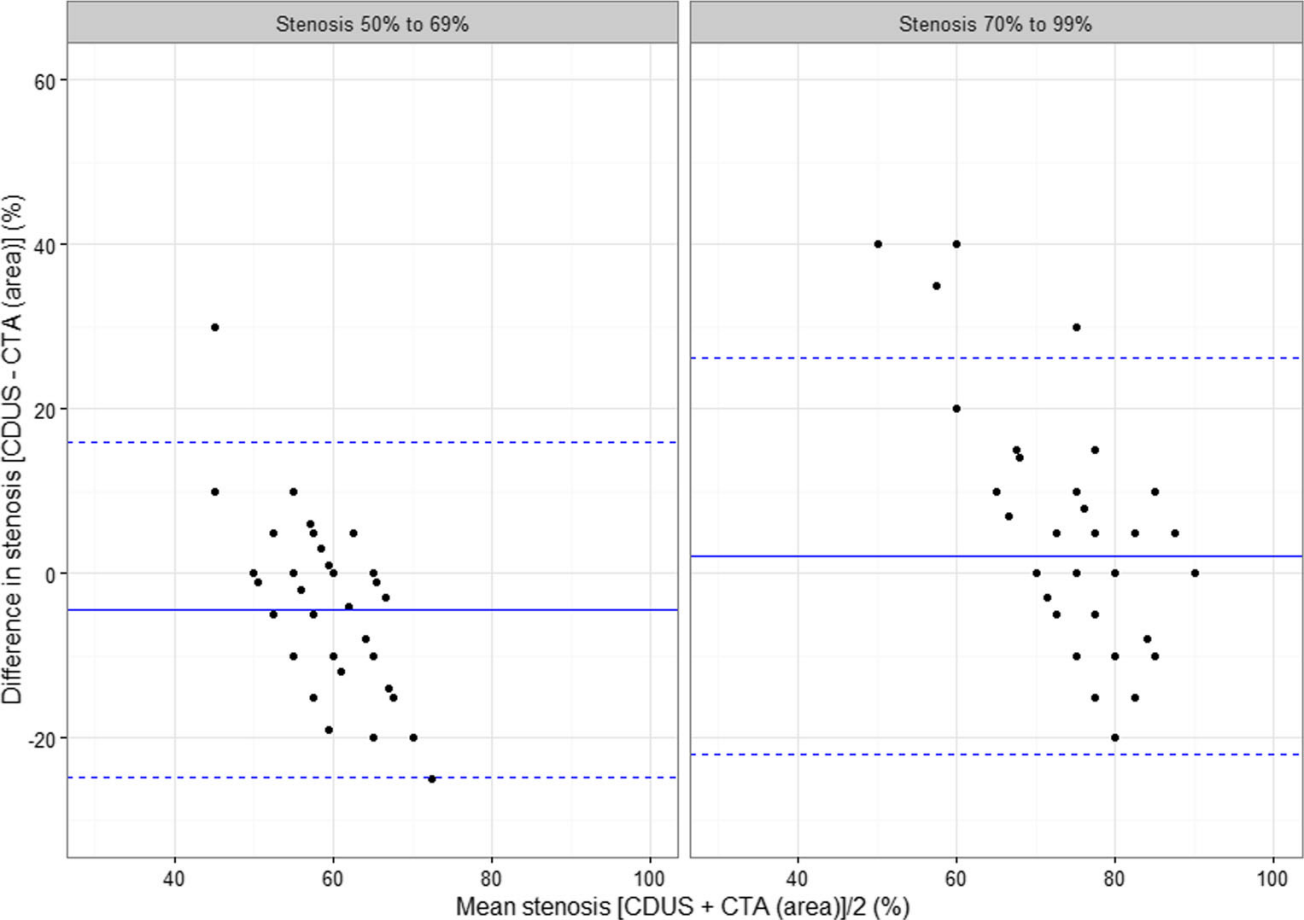
Bland-Altman plot of the difference between CDUS- and CTA (area)-based measurements

**Fig. 4:**
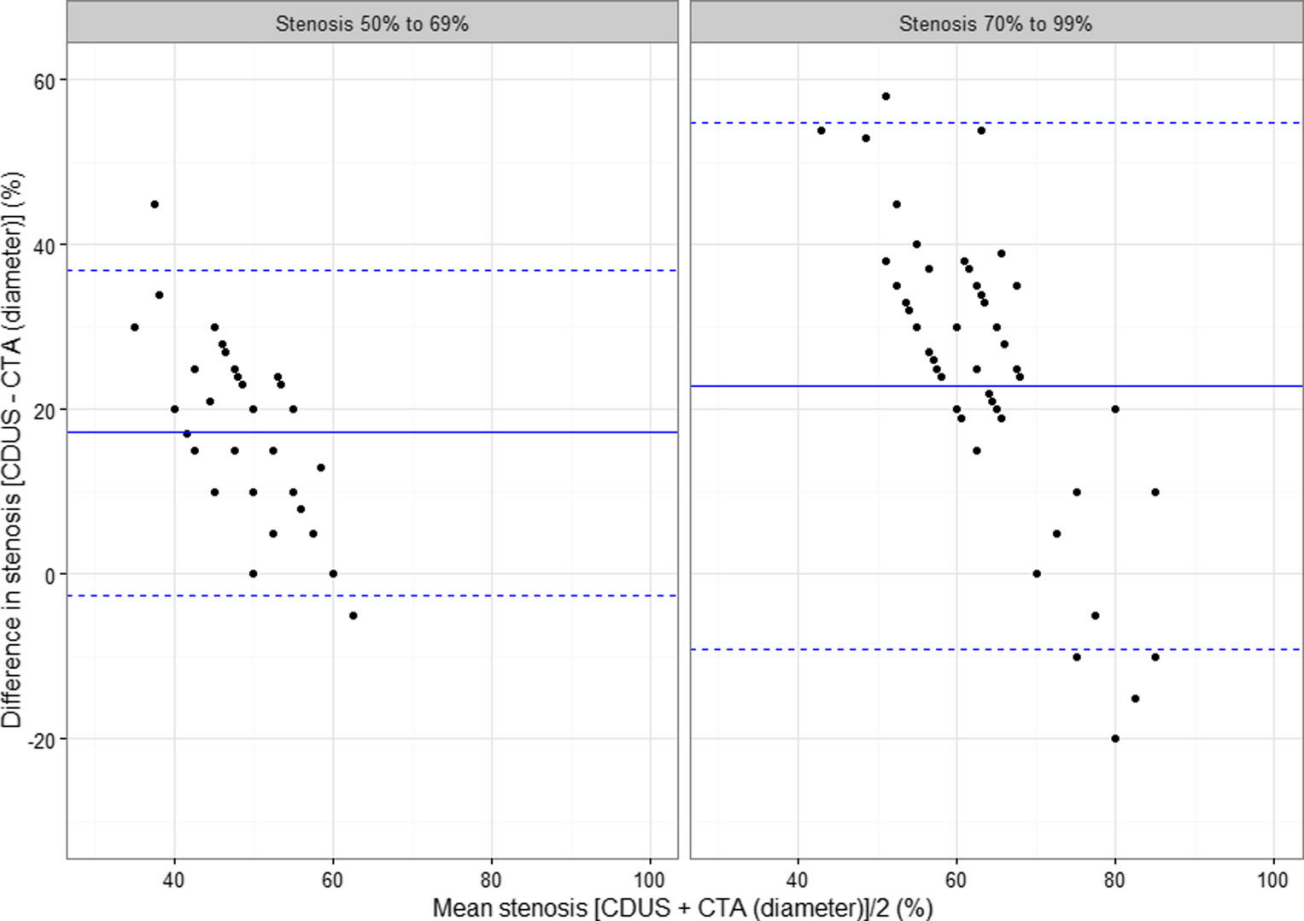
Bland-Altman plot of the difference between CDUS- and CTA (diameter)-based measurements

Figures 3 and 4 show Bland-Altman plots of differences between pairs of measurements against the mean of the same pair. The solid line is positioned at the mean difference for each stenosis group and for each of the two pairs, CDUS – CTA (area) and CDUS – CTA (diameter), while the two dotted lines mark two standard deviations around the mean, within which, assuming that differences are normally distributed, should be around 95% of the data. Without any association of the mean level and the magnitude of discordance between measurements, we would not expect to see any pattern in the scatter plot. According to the plots, the direction of the association is the same in both groups and for both pairs. For the CDUS-CTA (diameter) pair, the differences tend to spread from a large positive value for small mean stenosis of the pair, to negative values that are not as large for the greater mean stenosis. This means that the underestimation of stenosis by CTA (diameter) measurement was smaller for the higher stenosis level. For the CDUS-CTA (area) pair, the pattern is the same, but differences remain centered at 0.

When we look at Bland-Altman plot of differences between CDUS- and CTA (diameter)-based stenosis, we can see a large interval of two standard deviations (>30%) in the group of 70–99% stenosis. This means great differences in the absolute values of stenosis assessed with these two methods and, in a clinical sense, poor reliability when CTA (diameter) is used in selecting patients for surgery.

Table 2 juxtaposes characteristics of the predictive power of the two CT methods for detecting severe stenoses (70% or greater) requiring surgical intervention.

**Table 2:** Sensitivity, specificity, positive predictive value and negative predictive value of the CTA (area)- and CTA (diameter)-based measurements

Method	Sensitivity	Specificity	PPV	NPV
CTA (area)	0.81	0.77	0.84	0.72
CTA (diameter)	0.23	1	1	0.45

**Fig. 5:**
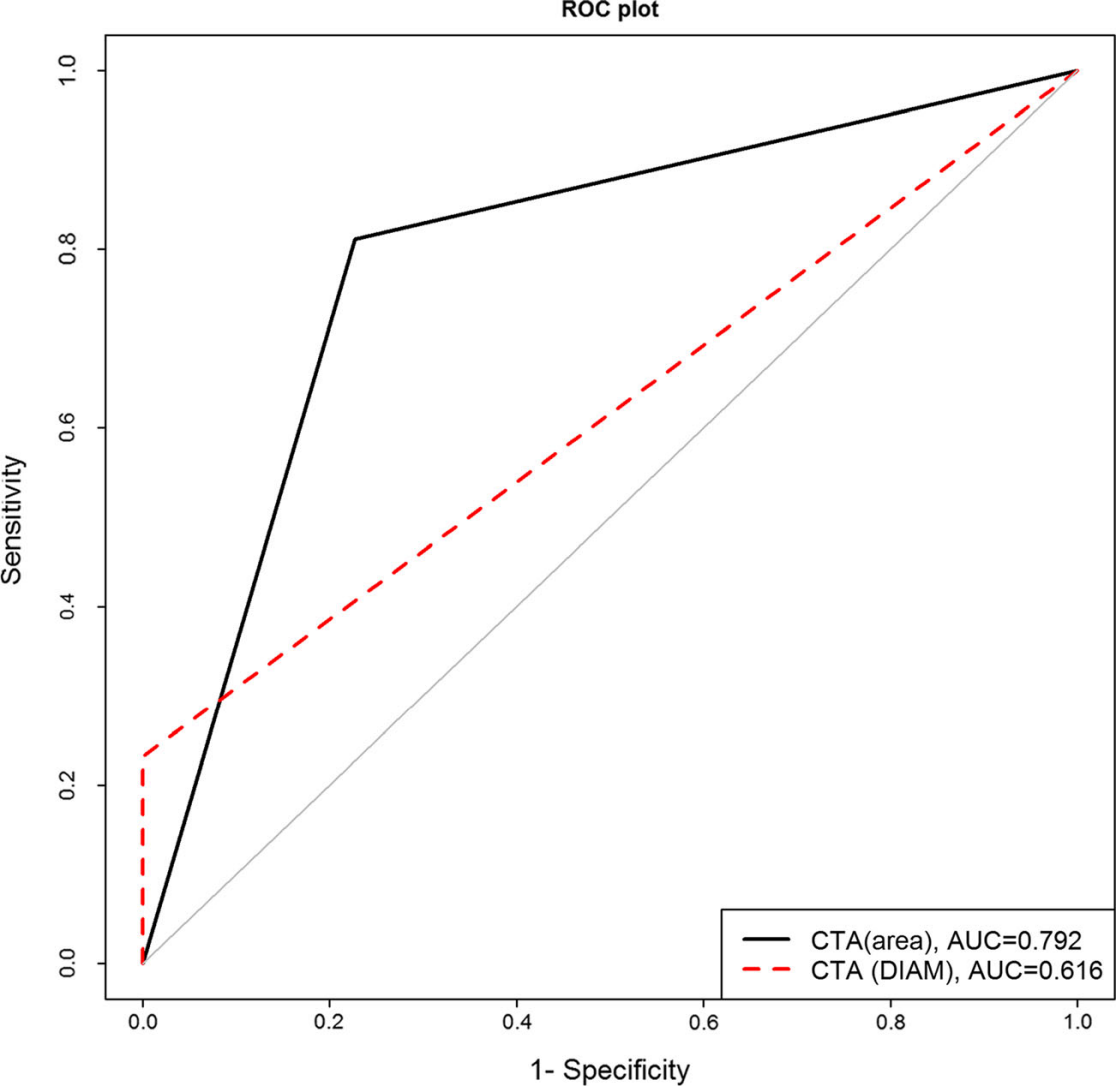
ROC curves demonstrating the specificity and sensitivity of both CTA (area) and CTA (diameter) methods in identifying surgical stenosis

Figure 5 shows the estimation of the ROC curves with respect to a clinically relevant question about the necessity of surgical intervention (Is surgery necessary? YES/NO).

The sensitivity and specificity of the CTA (area) method for detecting severe stenosis were well balanced, at 81% and 77% respectively. The sensitivity of CTA (diameter) was low, only 23%, while the specificity was perfect, resulting in 100%. This perfect specificity of the CTA (diameter) method is not surprising, since it shows systematic underestimation of the vessel stenosis, so all patients who did not need surgical intervention were classified as such by this method.

The area under the curve (AUC) of CTA (diameter) was 0.62 (0.57, 0.66), while the AUC from the ROC based on CTA (area) was 0.79 (0.71–0.87). The test of equality for the two AUCs resulted in a very small *p*-value (<0.0001), so we could reject the null hypothesis that the predictive power of the two CTA-derived methods (as characterized by AUC) is the same up to the level of 0.05. The predictive power of the CTA (area) method is therefore significantly higher than that of CTA (diameter) when it comes to correctly classifying vessels needing surgical intervention.

## Discussion

The use of cross-sectional area in assessing carotid stenosis with CTA is still being studied in relation to other methods. To evaluate the validity of two CTA methods, we used Bland-Altman analysis, measuring the absolute differences in the estimated level of stenosis, to determine the measurement error of the two methods in comparison with CDUS. We evaluated the magnitude and the direction of the bias from the two CTA methods with respect to CDUS measurements of the same vessels. CTA (area) method in the whole sample and in the group of surgical stenoses showed no significant bias in relation to CDUS measurements. In the group of 50–69% stenosis, this bias was significantly different from 0 in the negative direction (−4.5), but the *p*-value was not very small, meaning that the CTA (area) method in this group on average slightly overestimates the true stenosis level. CTA (diameter) method showed significant average bias in the whole sample and in both groups defined by the necessity of surgical intervention. The bias was between 17.3% and 22.9%, which means that this method clearly underestimates the degree of stenosis across the entire stenosis range. This discrepancy in stenosis grades is clinically considerable and the decision for surgery could be significantly altered if we rely only on CTA (diameter) measurements.

The predictive power of the CTA (area) method for detecting surgical stenoses, described by its ROC curve, was significantly higher than that of the CTA (diameter) method, with better balanced sensitivity and specificity and significantly higher AUC values.

The results of this study were not unexpected for us. Traditionally, the narrowest diameter of the lumen was used for calculation of carotid artery stenosis, because on DSA images only a diameter can be measured. With the appearance of CTA, the measurement of the area as a base for the calculation of stenosis has become possible. In theory, the measurement of stenosis based on cross-sectional area should be more accurate. Calculation according to diameter takes the narrowest diameter for evaluation and is based on the hypothesis that there is no stenosis in any other direction. Opposite of that, calculation according to area includes degree of stenosis in all directions. Due to eccentrically positioned plaques with ulcerations, the area of the residual lumen is often asymmetric and irregular. Therefore it is very important to consider all diameters of a stenotic segment when we calculate the degree of stenosis.

Other authors most often used correlation coefficients to evaluate the validity of these two CTA methods. Carnicelli et al. tested the accuracy of CTA using CDUS as a surrogate for true stenosis, and came to the conclusion that there is no significant difference between diameter and area measurements [6]. Bartlett et al., testing diameter and area measurements, concluded that carotid stenosis quantification based upon the narrowest diameter reliably predicts the more precise area measurements [7]. Van Prehn et al., comparing CTA stenosis grading with ultrasound, found that area measurements yielded correlation coefficients similar to those of diameter measurements, and concluded that diameter is an adequate approximation for area [8].

On the other hand, Zhang et al., testing diagnostic agreement between CTA and DSA, found that only satisfactory agreement was obtained between area stenosis on CTA and diameter stenosis on DSA, with lower correlation coefficients between CTA diameter and CTA area in stenoses with extremely non-circular lumen, compared with stenoses with circular lumen [9]. Similarly, Bucek et al. found a good correlation between CTA area and the results of DSA with superior inter-observer agreement compared to CTA diameter measurements [10].

In this study we found a higher accuracy with the CTA area method in the assessment of carotid stenosis, compared with CTA diameter, and our results are more concordant with the results of the last group of authors.

This study has several limitations. First, the sample size was relatively small, so that with a larger number of patients involved, the contribution of the study would be higher.

Second, we compared two CTA methods with CDUS, not with DSA, the gold standard in measurement of stenosis. In our institution DSA is not part of a routine diagnostic workup in the preoperative evaluation of carotid stenosis, and patients in our sample did not have DSA. However, CDUS has been rigorously tested against DSA during its many years of use, and we believe that it can serve as a surrogate for the true gold standard. In addition, DSA suffers from some limitations. It is a biplanar examination without the possibility of dynamic multiplanar vessel assessment, and it does not provide information on vessel wall and plaque composition.

Third, the CTA (area) and CTA (diameter) measurements were performed with commercially available AVA software, but we have no data on studies where its use is validated, which could potentially have an impact on the results.

## Conclusion

When CTA is used to quantify the degree of carotid stenosis, we suggest the use of an automatic calculation of stenosis based on cross-sectional area rather than on the narrowest diameter, because area considers asymmetric shapes of stenosis, while diameter depends on the projection. This recommendation is supported by the higher predictive power of the CTA area-based method for the correct classification of stenosis and a lack of a significant average difference in the grading of stenosis in all patients and in the group of surgical stenosis in relation to CDUS.
